# The brain regulatory program predates central nervous system evolution

**DOI:** 10.1038/s41598-023-35721-4

**Published:** 2023-05-27

**Authors:** Dylan Faltine-Gonzalez, Jamie Havrilak, Michael J. Layden

**Affiliations:** Department of Biological Sciences, Lehigh University, Bethlehem, PA USA

**Keywords:** Neurogenesis, Evolutionary developmental biology

## Abstract

Understanding how brains evolved is critical to determine the origin(s) of centralized nervous systems. Brains are patterned along their anteroposterior axis by stripes of gene expression that appear to be conserved, suggesting brains are homologous. However, the striped expression is also part of the deeply conserved anteroposterior axial program. An emerging hypothesis is that similarities in brain patterning are convergent, arising through the repeated co-option of axial programs. To resolve whether shared brain neuronal programs likely reflect convergence or homology, we investigated the evolution of axial programs in neurogenesis. We show that the bilaterian anteroposterior program patterns the nerve net of the cnidarian *Nematostella* along the oral-aboral axis arguing that anteroposterior programs regionalized developing nervous systems in the cnidarian–bilaterian common ancestor prior to the emergence of brains. This finding rejects shared patterning as sufficient evidence to support brain homology and provides functional support for the plausibility that axial programs could be co-opted if nervous systems centralized in multiple lineages.

## Introduction

Efforts to identify the origin(s) of bilaterian CNSs have been reinvigorated by advances in genomics, phylogenetics, and increased taxon sampling^1^. CNSs are typically organized along the anteroposterior axis, with an anterior brain and lateral nerve cords that extend posteriorly from the brain. Determining the homology of nerve cords is challenging, because they are not patterned by a clearly conserved molecular program nor do they emerge from a clearly homologous position on the bilaterian bauplan^2,3^. These observations suggest that understanding the origin of brains will be critical to determine the origin of CNSs.

**Figure 1:**
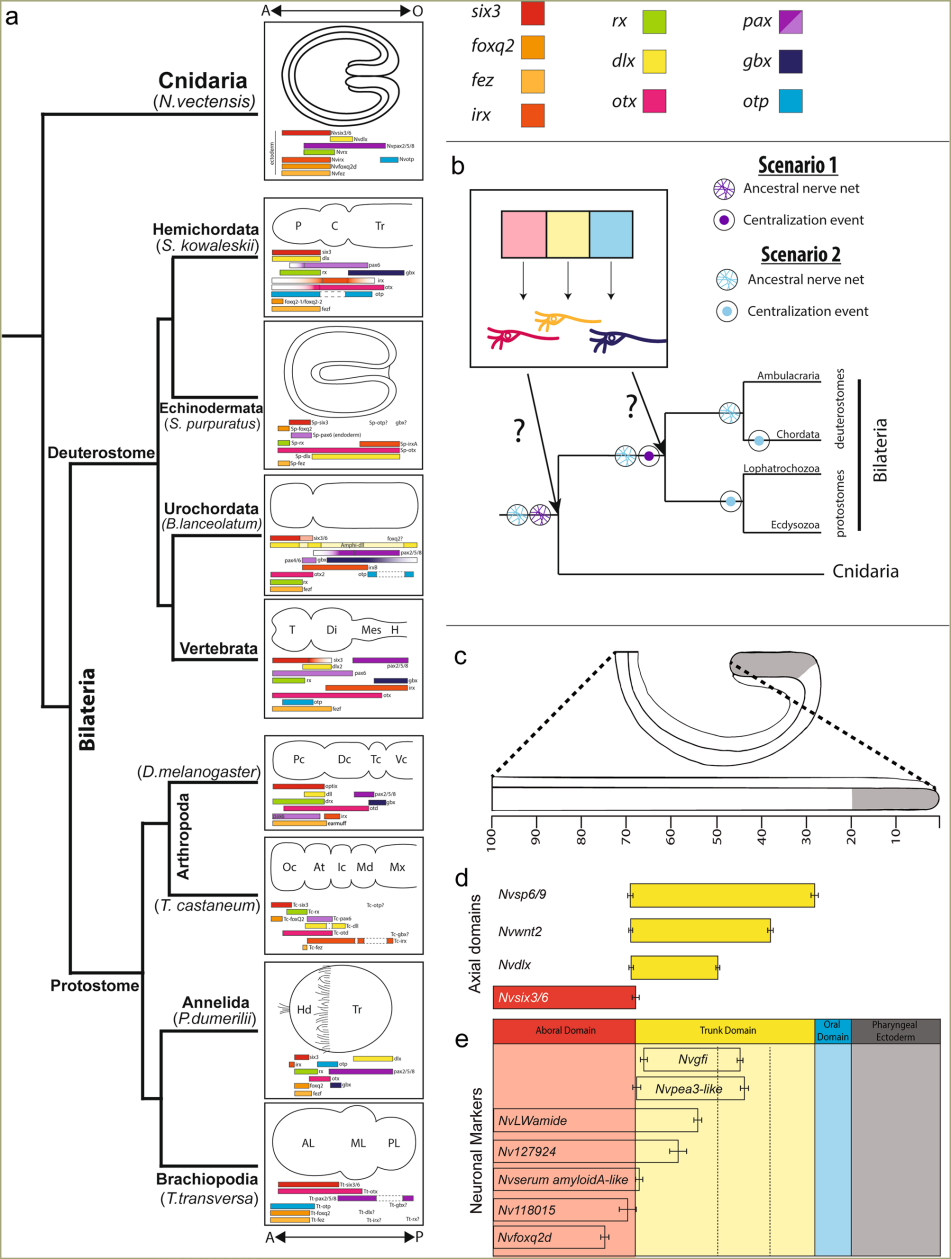
Stripes of regionalized gene expression pattern developing nervous systems. (**a**) Published expression domains of regional gene homologs shown in representative taxa from bilaterian species and *Nematostella*. (**b**) Strips of regionalized gene expression regulate neuronal fates within their respective domain (box). This patterning mechanism may have evolved coincidently with the evolution of an ancestral CNS (Scenario 1) or prior to CNS evolution (Scenario 2). (**c**) Positional information was normalized to percent embryo length with 0% being the tip of pharyngeal ectoderm (grey) and 100% at the aboral pole. (**d**) Average boundaries of candidate neuronal subtype regulators tested in this study. Example data in Supplemental Fig. 1. (**e**) Average expression domain for neuronal subtype markers. Example data in Supplemental Fig. 1. The bars indicate the 95% confidence interval for each domain boundary. Full list of homologs and their associated references can be found in Table S1.

Initially, the presence of tripartite brains patterned by strikingly similar mechanisms in *Drosophila* (protostome) and vertebrates (deuterostome) suggested that a morphologically complex brain arose deep in the bilaterian lineage and was considered strong support for the homology of all brains. Fossil evidence and careful mapping of morphologies onto the phylogeny argue morphological complexity is convergent^2–6^. However, similar gene expression patterns along the anteroposterior axis appear well conserved regardless of brain morphology (Fig. 1a)^4,5,7–12^. In the ectoderm, presumptive brains are regionalized by a posterior source of canonical Wnt (cWnt) and other morphogens^13–16^. Regionalization generates stripes of gene expression that partition the anteroposterior axis into distinct domains that in turn give rise to distinct neuronal subtypes (Fig. 1a,b(box)). For example, *six3* is a highly conserved anterior region marker that promotes anterior neuronal fates and represses posteriorizing cWnt (Fig. 1a)^12,17^. The remaining regionalized genes (*e.g. irx, foxq2, rx, otx, gbx, pax, otp, fez, dlx*) are expressed in similar, albeit not identical, patterns (Fig. 1a)^5,8,18–33^. This similarity of gene expression between regionalization programs in protostome and deuterostome brains is considered strong support for their homology (Fig. 1b; Scenario 1)^4,5^.

Interrogation of “brain” programs in additional species suggests that the conserved anteroposterior patterning program is not sufficient to reflect homology. Regionalized genes broadly pattern the ectoderm of hemichordates, echinoderms, brachiopods, and cnidarians, all of which lack centralized nervous systems and/or brain-like anterior neuronal condensations (Fig. 1a)^8,19,29,31^. Even within species that have a brain, stripes of regional genes are not restricted to the neuroectoderm^3,22,32–36^. This has led to the proposal that axial patterning programs have been co-opted to pattern the brain^2^. To understand the significance of similar regionalization programs with regards to brain evolution, it is critical to determine when the neuronal role for the axial programs evolved. For conserved regionalization programs to provide strong support for homology, their neuronal function should coincide with the emergence of a single ancestral brain, when the single centralization event occurred, presumably in the protostome–deuterostome ancestor (Fig. 1b, Scenario B). However, if axial patterning genes functioned to regionalize ancestral nerve nets, it would argue that one ancestral function of axial programs genes was to pattern developing nervous systems, which would preclude the use of this trait alone as support for a common origin of all bilaterian brains. Thus, we set out to identify when axial programs evolved a role in regionalized patterning of developing nervous systems.

Cnidarians (e.g. sea anemones, corals, jellyfish, hydras) are the sister taxon to bilaterians and previous efforts demonstrated that cnidarian nerve nets and bilaterian centralized nervous systems evolved from a nerve net-like nervous system in their common ancestor (Fig. 1b)^37^. This makes cnidarians informative for determining if neuronal roles for axial programs evolved prior to or within the bilaterian lineage. Graded cWnt activity highest at the oral end of the cnidarian sea anemone *Nematostella vectensis* patterns homologs of bilaterian regionally expressed genes into stripes along oral–aboral (O–A) axis using the same regulatory logic observed in bilaterian anteroposterior axis^38,39^. Moreover, homologs are expressed in the same relative order in both bilaterians and *Nematostella*^38–43^ (Fig. 1a). These data indicate that O–A patterning in cnidarians and A–P patterning in bilaterians are derived from a common ancestral axial program^38^. Although the full complement of *Nematostella* neuronal subtypes is poorly understood, expression of the few known subtype markers indicate that the nerve net is regionalized along the O–A axis during development^44,45^*.* Similarly, global misexpression of neuronal transcription factors are only able to increase expression of target genes within the domains in which they are normally expressed^45^. These observations argue that axial programs impinge on neuronal programs to pattern neuronal subtypes in a regionalized manner along the O–A axis. Here we test the hypothesis that homologs of the bilaterian anteroposterior regionalization program regulate neuronal patterning along the O–A axis in *Nematostella.*

## Results

### Identifying candidate neuronal subtype specifiers

**Figure 2:**
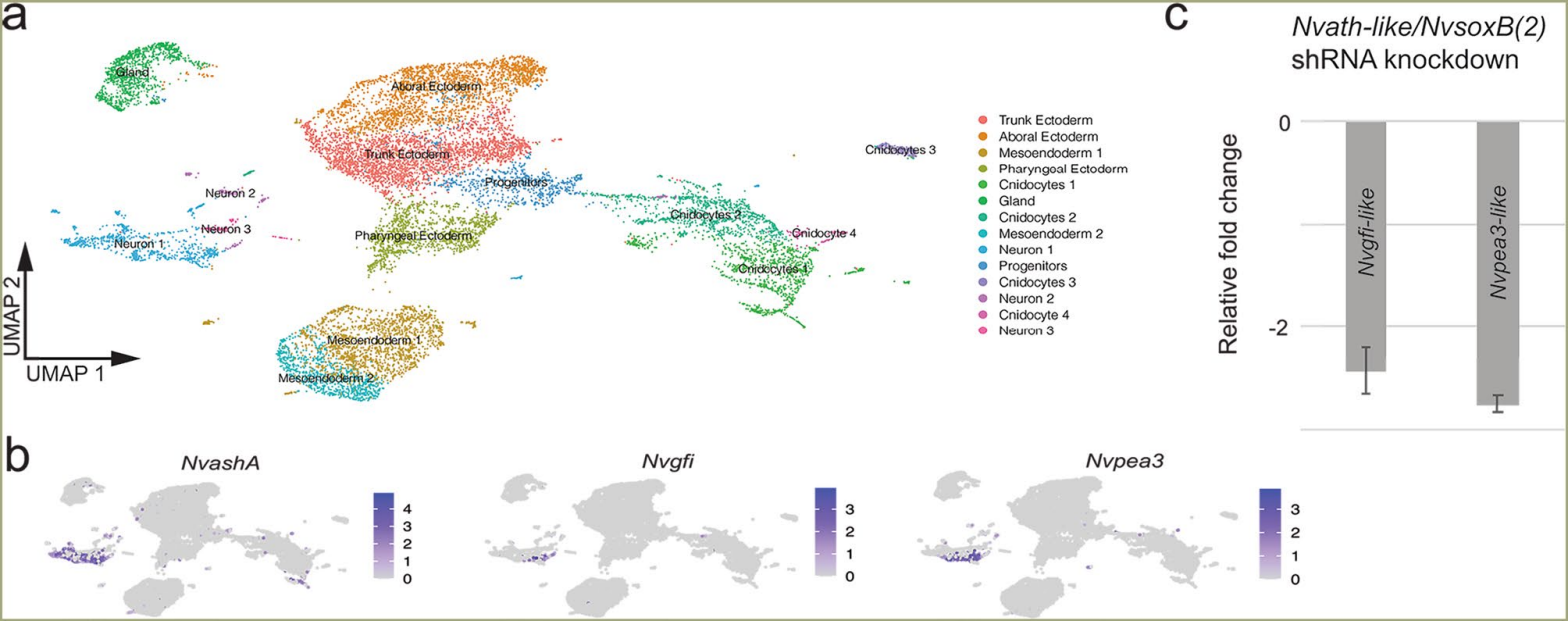
*Nvpea3-like* and *Nvgfi-like* are neuronal subtype identifiers. (**a**) Cell clusters identified in single cell RNA sequencing data from late gastrula embryos. Cluster identities were assigned as previously described (Table S2)^47^. (**b**) The known neuronal markers *NvashA, Nvgfi-like,* and *Nvpea3-like* cluster within the identified neuronal cluster. (**c**) *Nvgfi-like* and *Nvpea3-like* expression is reduced when known neuronal genes *Nvath-like* and *NvsoxB(2)* are knocked down using shRNA mediated gene knockdown*.*

To identify putative neuronal regionalization genes and regionalized neuronal subtypes, the expression domain of regional genes and neuronal markers were quantified in gastrulae by calculating the oral and aboral expression limits for each gene expressed as percent embryo length (PEL) (Fig. 1c–e; Supplementary Fig. 1) (see “Methods”). This approach confirmed the known boundary between the *Nvsix3/6*+ aboral domain and the *Nvwnt2*+ domain^38^, and the expression of *Nvfoxq2d* within the *Nvsix3/6*+ aboral domain (Fig. 1c–e; Supplementary Fig. 1a,b)^46^. The previously identified neuronal genes *Nv118015* and *Nvserum amyloid A-like* were restricted to the aboral domain (Fig. 1d,e; Supplementary Fig. 1c,d). *Nv127924*+ and *NvLWamide-like*+ neurons are expressed throughout the aboral and the *Nvwnt2*+ and *Nvdlx*+ trunk domains (Fig. 1d,e; Supplementary Fig. 1e,f) making them less informative for this study. *Nvpea3-like* and *Nvgfi* have previously been identified as potential neuronal markers^44^. *Nvpea3-like* initiates at the boundary of trunk and aboral domains and spans the *Nvdlx*+ trunk region terminating within the *Nvwnt2*+ domain (Fig. 1d,e; Supplementary Fig. 1j). *Nvgfi-like* is also expressed in the trunk region within the *Nvdlx*+*, Nvwnt2*+*,* and *Nvsp6-9*+ domains (Fig. 1d,e; Supplementary Fig. 1i). To confirm that *Nvgfi-like* and *Nvpea3-like* are neuronal markers, we showed that cells expressing each marker are localized to known neuronal clusters in our single cell RNAseq data generated from gastrula stage embryos (Fig. 2a,b), and that they both require *NvsoxB(2)* and *Nvath-like* for expression (Fig. 2c). These data suggested that we have identified neuronal subtypes born within the aboral domain and trunk domains and that *Nvsix3/6* and *Nvwnt2* are candidate regional genes upstream of neuronal patterning.

### *Nvsix3/6* specifies aboral neurons

**Figure 3:**
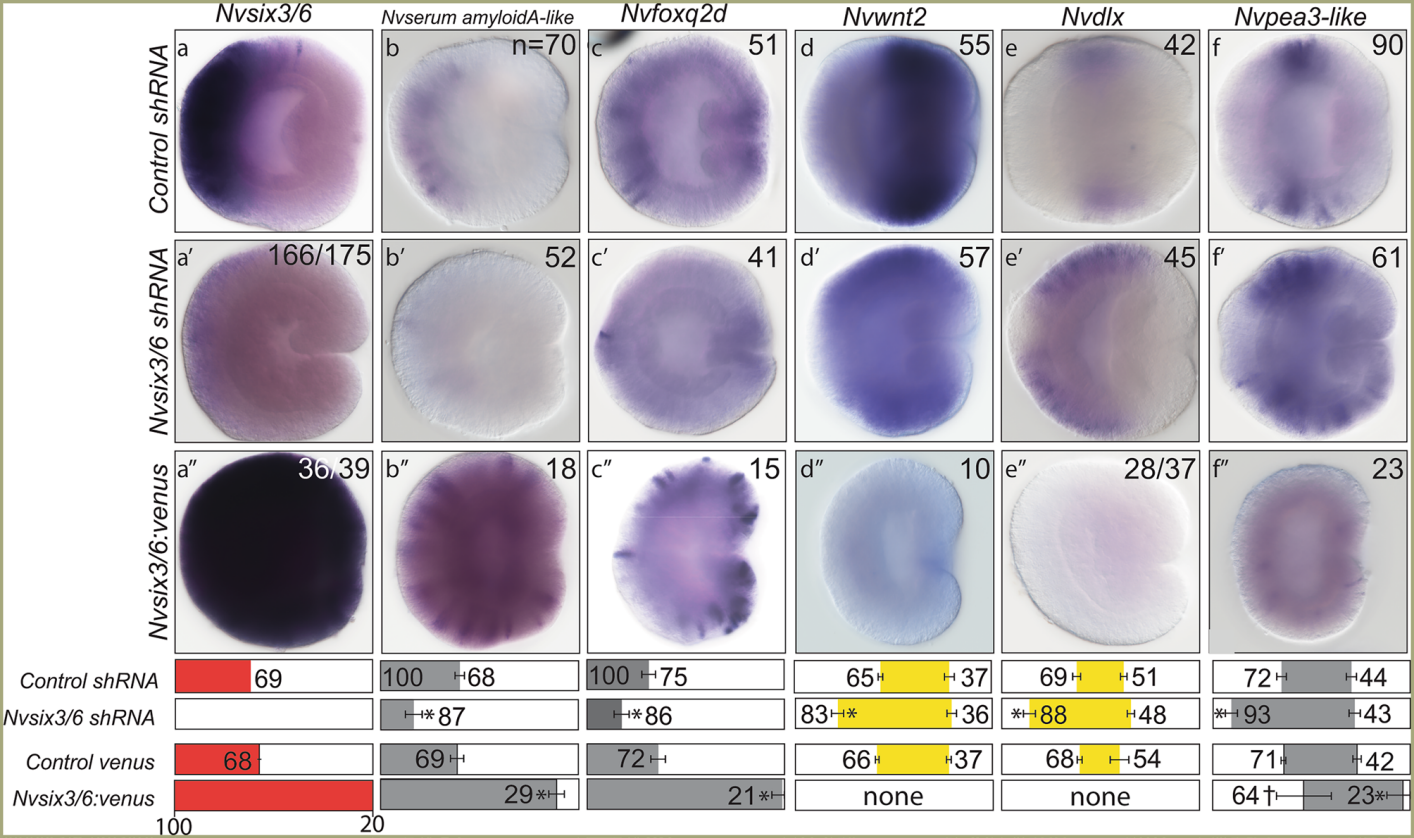
*Nvsix3/6* is *necessary and sufficient to promote aboral fates.* (**a–f**) Control sRNA injected animals. (**a′**–**f′**) *Nvsix3/6* shRNA injected animals. (**a″**–**f″**) *Nvsix3/6:venus* mRNA injected animals. In all images, aboral is to the left and oral is to the right. Quantification of percent embryo length (PEL) positions for the aboral (left-boundary) and oral (right-boundary) expression limits for each treatment and their control are shown below the images. Bars represent 95% confidence interval. * indicates that treated values are statistically different (p ≤ 0.05) from controls and that the 95% confidence intervals for the values do not overlap, and † indicates that treated values are statistically different (p ≤ 0.05) from controls, but that 95% confidence intervals overlap.

To determine if the *Nvsix3/6* regional gene patterns neurons generated within its expression domain, we tested the functional requirements of *Nvsix3/6* to pattern neurons born within its domain. shRNA mediated gene knockdown of *Nvsix3/6* resulted in the loss of aboral neurons. Aboral subtype markers *Nvserum amyloid A-like, Nvfoxq2d,* and *Nv118015* were undetectable in many injected animals. When present, their domain was severely reduced (compare Fig. 3a–c with Fig. 3a′–c′; Supplementary Fig. 2b,b′). Unlike the aboral restricted genes, *Nv127924* was reduced but not eliminated in *Nvsix3/6* shRNA injected animals (Supplementary Fig. 2a,a′), and *NvLWamide-like* expression was not noticeably impacted by loss of *Nvsix3/6* (Supplementary Fig. 2c,c′). Loss of aboral subtype markers was accompanied by an aboral expansion of trunk identity and trunk neuronal fates into the aboral domain, which mimics the *Nvsix3/6* morphant and increased cWnt phenotypes (Fig. 3d–f,d′–f″; Supplementary Fig. 3d,d′)^39,42,43^. Injection of *Nvsix3/6:venus* mRNA into single cell zygotes resulted in ubiquitous misexpression of *Nvsix3/6* (Fig. 3a″) and expanded the oral expression limit for all neuronal subtypes (Fig. 3b″,c″,f″; Supplementary Fig. 2a″–c″). Although the oral boundary of *Nvpea3-like* shifted orally, the overall expression of *Nvpea3-like* was severely reduced (Fig. 3f″). *Nvwnt2* and *Nvdlx* expression was undetectable in *Nvsix3/6* overexpressing animals (Fig. 3d″,e″; Supplemental Fig. 2d″). These findings suggest that *Nvsix3/6* is necessary and sufficient to promote aboral neuronal fates.

**Figure 4:**
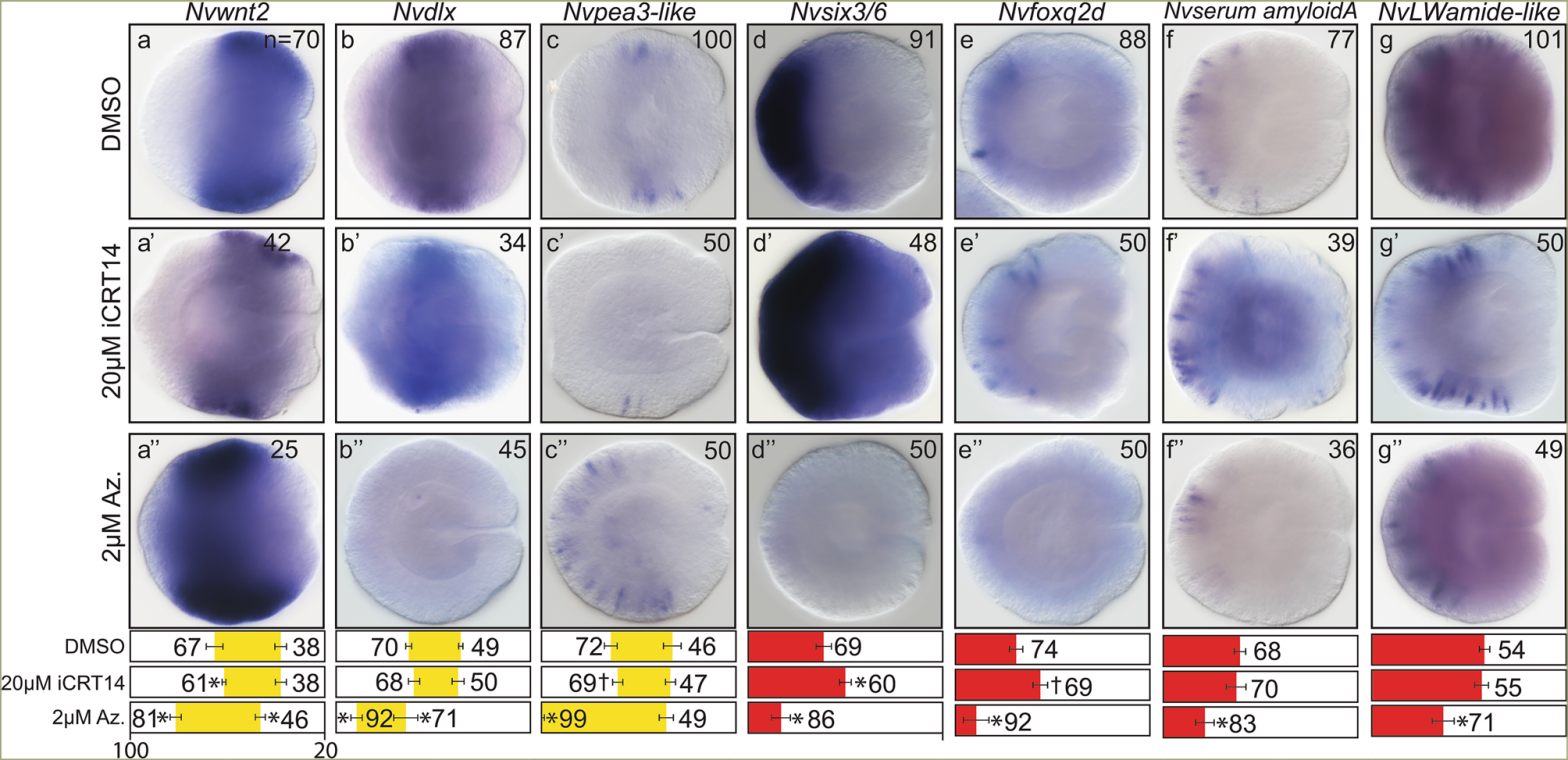
Manipulating cWnt levels alters regional and neuronal gene expression. (**a**–**g**) DMSO treated control. (**a′–g′**) Treatment with 20 µM iCRT14 from late blastula to gastrula stage. (**a**″**–g**″) Treatment with 2 µM Azenkenpaullone from late blastula to gastrula stage. In all images, aboral is to the left and oral is to the right Quantification of percent embryo length (PEL) positions for the aboral (left-boundary) and oral (right-boundary) expression limits for each treatment and their control are shown below. Bars represent 95% confidence interval. * indicates that treated values are statistically different (p ≤ 0.05) from controls and that the 95% confidence intervals for the values do not overlap, and † indicates that treated values are statistically different (p ≤ 0.05) from controls, but that 95% confidence intervals overlap.

*Nvsix3/6* is known to antagonize cWnt activity in the aboral domain and anterior brain^43^. To assess whether cWnt levels impact neuronal fates, resulting changes to neuronal fates were assessed after increasing or decreasing cWnt activity in gastrula stage animals (Fig. 4). Inhibition of cWnt with iCRT-14 phenocopied the previously reported oral expansion of *Nvsix3/6* and expanded aboral neuronal subtype expression orally (Fig. 4e′,f′)^43^. Similarly, the aboral boundary of the trunk domain gene *Nvwnt2* and trunk neuronal marker *Nvpea3-like* was shifted orally (Fig. 4a′,c′). Increased cWnt reduced the aboral domain and aboral neuronal subtype expression while expanding the aboral boundary of Trunk regional and *Nvpea3-like* expression, which was previously observed for trunk markers (compare Fig. 4a–g with Fig. 4a″–g″)^39,42,43^. Because changes in cWnt can both shift regional gene expression and neuronal fate patterning, it was not clear from this observation if *Nvsix3/6* in the aboral domain patterns neuronal fates by actively promoting aboral identity, simply repressing cWnt, or a combination of both, to activate aboral neuronal gene expression.

**Figure 5:**
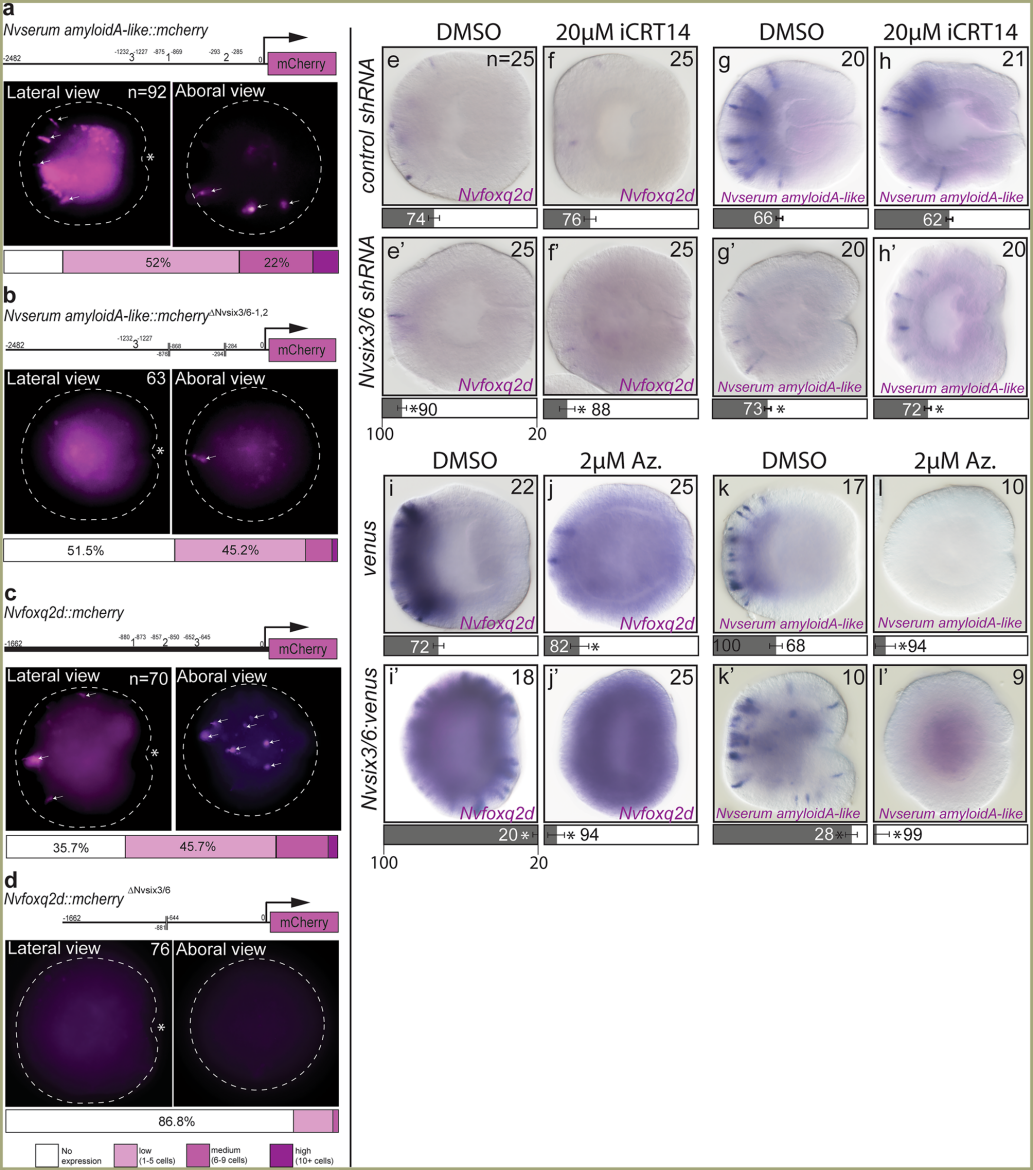
*Nvsix3/6* activates aboral gene expression. (**a**,**b**) *Nvserum-amyloid A-like::mcherry* transgenes were tested for expression in F0 animals. Wild-type (**a**) shows aboral expression, which is severely reduced in enhancers lacking *Nvsix3/6* binding sites (**b**). (**c**,**d**) *Nvfoxq2d::mcherry* transgenes were tested for expression in F0 animals. Wild-type (**c**) shows aboral expression, which is severely reduced in enhancers lacking *Nvsix3/6* binding sites (**d**). (**e**–**h**) The impact of iCRT14 treatments on aboral gene expression in uninjected animals (**e**,**g**) or in animals injected with *Nvsix3/6* shRNA (**e′**,**g′**) compared to controls (**d**,**f**). (**i–k**) The impact of Azankenpaullone treatments on aboral gene expression in uninjected animals (**i**,**l**) or in animals injected with *Nvsix3/6* mRNA (**i′**,**k′**) compared to controls (**h**–**j**). Bars represent 95% confidence interval. * indicates that treated values are statistically different (p ≤ 0.05) from controls and that the 95% confidence intervals for the values do not overlap, and † indicates that treated values are statistically different (p ≤ 0.05) from controls, but that 95% confidence intervals overlap. In (**a–d**) embryos are outlined by dotted line, oral in lateral views is indicated with an asterisk, and neurons expressing the transgene are indicated by white arrows.

To determine aboral neuronal fates are patterned by *Nvsix3/6* or the changes to cWnt levels that follow disruptions to *Nvsix3/6* levels, we sought to clarify *Nvsix3/6s* role in aboral neuronal patterning. First, we tested the possibility that *Nvsix3/6* may function directly on neuronal genes. Enhancer regions for both *Nvserum amyloid-A* like and *Nvfoxq2d* were previously identified, and both enhancers contain 3 putative *Nvsix3/6* binding sites (Fig. 5). To determine the requirement of *Nvsix3/6* for expression of each transgenes, we compared the ability of wild type (Fig. 5a,c) and enhancer fragments lacking *Nvsix3/6* sites (Fig. 5b,d) to drive *mcherry* expression in F0 animals. The number of neurons and percentage of animals with expression of the transgene in the aboral domain were quantified. The wild type *Nvserum amyloid-A like* fragment had aboral expression in ~ 75% of the animals with ~ 25% of animals exhibiting medium to high levels of expression (Fig. 5a). Deleting two of the three *Nvsix3/6* sites resulted in expression in less than half of the animals, with only 3% of the animals having medium to high expression (Fig. 5b). ~ 65% of wild type *Nvfoxq2d* enhancers showed expression (Fig. 5c), whereas 87% of animals injected with enhancer fragments lacking the three *Nvsix3/6* binding sites had no detectable expression (Fig. 5d). Additionally, we reasoned that if low cWnt levels played a more significant role than *Nvsix3/6* to promote aboral neuronal fates, then treating *Nvsix3/6* knockdown animals with the cWnt antagonist iCRT14 should at least partially rescue the reduction of aboral neuronal fates. Treatment of *Nvsix3/6* shRNA injected animals with 20 µM iCRT14 did not rescue either *Nvserum amyloid A-like* or *Nvfoxq2d* expression (Fig. 5f,f′–h,h′). Thus, we conclude that *Nvsix3/6* actively promotes aboral neuronal fates independently of its role repressing cWnt activity.

Additionally, we tested whether *Nvsix3/6* repression of cWnt was required for aboral neuronal fates. This was accomplished by determining if *Nvsix3/6* was sufficient to rescue the aboral identity in animals treated with the cWnt agonist Azankenpaullone Fig. 5j,i). *Nvsix3/6* misexpressing animals treated with 2 µM of Azankenpaullone failed to rescue aboral fates (Fig. 5j′,l′). Overall, the phenotypes resembled the Azankenpaullone only treatments (compare Fig. 5j′ and l′ with Fig. 4e″ and f″) suggesting that either high levels of cWnt, a trunk regional marker activated by cWnt, or both repress aboral neuronal fates. Collectively, these findings suggest that *Nvsix3/6* promotes aboral fates by promoting expression of aboral neuronal subtype markers and by inhibiting trunk identity through repression of cWnt.

### *Nvwnt2* promotes trunk and represses aboral neuronal subtype identity

**Figure 6:**
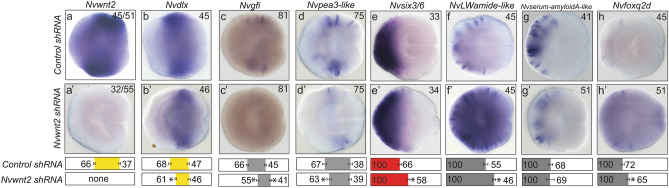
*Nvwnt2* promotes trunk identity and suppresses aboral fates. (**a**–**h**) Control sRNA injected animals. (**a′–h′**) *Nvwnt2* shRNA injected animals. In all images, aboral is to the left and oral is to the right. Quantification of percent embryo length (PEL) positions for the aboral (left-boundary) and oral (right-boundary) expression limits for each treatment and their control are shown below the images. Bars represent 95% confidence interval. * indicates that treated values are statistically different (p ≤ 0.05) from controls and that the 95% confidence intervals for the values do not overlap, and † indicates that treated values are statistically different (p ≤ 0.05) from controls, but that 95% confidence intervals overlap.

To gain insights about the neuronal role of trunk genes, we reduced *Nvwnt2* and *Nvdlx expression* with shRNA mediated knockdown (Fig. 6). As expected, the loss of *Nvdlx* did not disrupt expression of regional genes or neuronal markers along the O-A axis (Supplemental Fig. 3a,a′–h,h′). Reduction of *Nvwnt2* resulted in an oral shift in the aboral boundary of *Nvdlx* (Fig. 6a,a′). The aboral boundary of *Nvpea3-like* and *Nvgfi-like* expression shifted orally and the overall expression levels and domain sizes were reduced for both genes (Fig. 6c,c′,d,d′). The oral boundary of *Nvgfi-like also* shifted orally, which was not observed for *Nvpea3-like* (Fig. 6c′)*.* The reduced expression levels for *Nvpea3-like* resemble the decreased expression observed in iCRT14 treated embryos (Fig. 4c′). Aboral domain gene *Nvsix3/6* and neuronal markers *Nvfoxq2d* and *NvLWamide-like* all expanded their expression orally in *Nvwnt2* knockdown animals (Fig. 6e,e′–h,h′). *NvsoxB(2)* and *Nvath-like,* neural progenitor markers (*52*, *53*), were not reduced in *Nvwnt2* shRNA injected animals (Supplementary Fig. 3i) indicating that loss of *Nvpea3-like* and *Nvgfi-like* is not due to a reduction in neurogenesis but rather from changes to patterning. These observations suggest *Nvwnt2* promotes the trunk neuronal genes *Nvpea3-like* and *Nvgfi-like* and represses the aboral fates.

## Discussion

### A model for nerve net regionalization along the O–A axis

**Figure 7:**
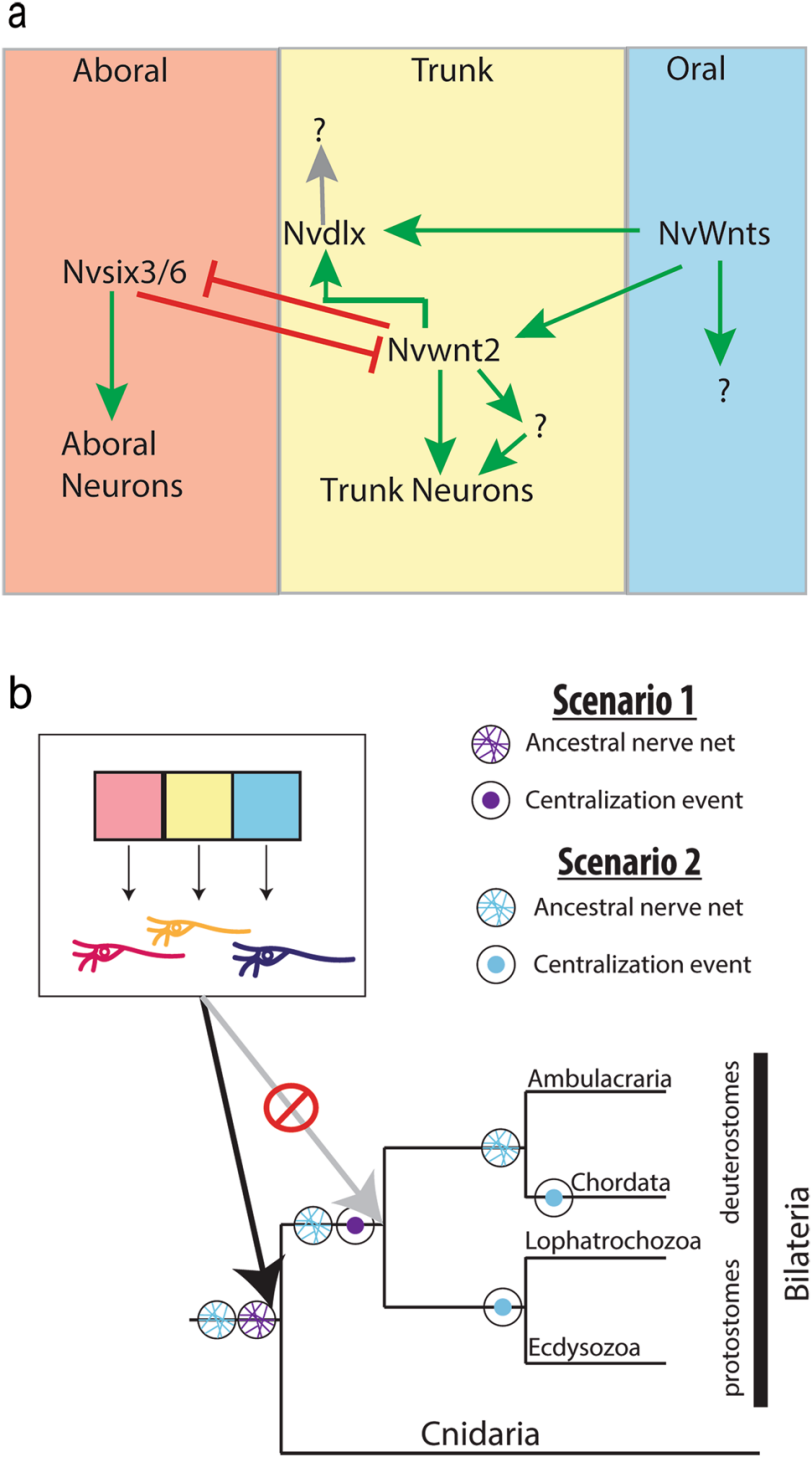
Model of *Nematostella* nerve net patterning and evolution of neuronal regionalization programs. (**a**) Schematic describing how regionalized genes pattern the developing *Nematostella* nerve net. (**b**) Regional patterning of nervous systems evolved in the cnidarian–bilaterian ancestor (black arrow), not within the bilaterians (grey arrow), suggesting that shared regionalization programs support both scenarios for CNS evolution.

We propose that neuronal fates are patterned along the O–A axis in *Nematostella* by stripes of regionalized gene expression that are established in part by graded cWnt activity at the oral end (Fig. 7a). The oral cWnt is opposed by *Nvsix3/6* and FGF activity at the aboral end^38,39,42,43^. Regionally restricted domain genes both promote neuronal fates born within their domain and prevent expression of adjacent domain genes and neuronal fates (Fig. 7a). Specifically, we show that the aboral domain gene *Nvsix3/6* promotes aboral fates by activating expression of *Nvserum amyloid A-like* and *Nvfoxq2d* while simultaneously repressing trunk identity. Similarly, within the Trunk region, *Nvwnt2* promotes the expression of *Nvpea3-like* and *Nvgfi-like* and represses *Nvsix3/6* and *Nvfoxq2d* aboral fates. Additional factors must regulate Trunk patterning, as there are a number of regionally expressed genes throughout the Trunk that likely generate multiple molecularly distinct domains^39^. Additional efforts to identify neuronal fates downstream of regionally expressed genes will be necessary to fully describe nerve net patterning. In addition to inputs from regionally expressed genes, the level of cWnt may also influence neuronal patterning directly. For example, *NvLWamide-like* is disrupted by pharmacological manipulation of cWnt activity and in animals with reduced *Nvwnt2,* but not by loss of *Nvsix3/6.* However*,* we cannot rule out that cWnt is acting through a yet to be identified regionally expressed gene(s) that span multiple domains or that *NvLWamide-like*+ cells are comprised of multiple sub-populations each restricted to a distinct domain*.*

### Implications for understanding nervous system evolution

Work in *Hydra* polyps suggest that their nerve net is also regionalized. When coupled with our data it suggests that the nerve net in the cnidarian-bilaterian ancestor was regionalized. Thus, whether brains evolved once or multiple times, they emerged from a regionalized ancestral nerve net. Our data demonstrate that the mechanism that regionalizes bilaterian brains predates bilaterian divergence and CNS evolution. The stripes of gene expression are conserved between bilaterians and *Nematostella* (Fig. 1)*,* and regionalized genes regulate neuronal fates born within their respective domains. The similarities in *Nematostella* nerve net and brain patterning along the O–A and A–P axes argue that they stem from a shared ancestral program (Fig. 7b). Because regionalization programs broadly pattern the ectoderm in both cnidarians and bilaterians and brain/CNSs are derived from ectodermal tissue, it is reasonable to expect that regionalized gene expression would be maintained as nervous systems centralized within the ectoderm. At a minimum our findings reject the argument that conserved regionalization programs are sufficient to support the homology of bilaterian brains. Our findings support the plausibility of the co-option hypothesis because no novel function would need to evolve for axial programs to be independently co-opted.

The question remains, how do we determine if bilaterian brains, and by extension CNSs, evolved once or multiple times within the Bilateria? We speculate that at least two additional areas of study will contribute to our understanding about the origin(s) of brains and CNSs. First, identifying the mechanisms that induce brain/CNS formation in phylogenetically informative bilaterian taxa will likely have a large impact on this debate. CNS development is distinct from nerve net development in that a continuous portion(s) of the ectoderm is induced to adopt a neuronal fate. It is no longer clear that BMP inhibition is a deeply conserved component of induction, as work in lophotrochozoans, hemichordates, and echinoderms offer conflicting views of the role BMP plays in neuronal specification^48–51^. Thus, additional efforts to understand the origin(s) and evolution of neural induction are necessary to determine if induction uses conserved or divergent mechanisms across taxa. Highly divergent induction programs would support the multi-origin hypothesis, whereas a deeply conserved inductive mechanism would support a single origin for brains/CNSs. Second, efforts to better understand the regulatory networks that underly regionalization programs will likely prove important to resolving whether brains/CNSs evolved once or multiple times. Initial expression of regionalized genes downstream of morphogens has been relatively well studied, but little efforts have been put into the determining the regulatory networks that act to maintain regionalization in the neural ectoderm during brain development, or in the programs downstream of regional genes that promote neuronal subtype patterning within the unique domains generated along the A–P axis of developing brains. The added value of the regulatory networks is that it provides mechanistic data that increase our ability to quantify how similar or dissimilar regionalization programs in developing brains are. This analysis may identify key changes to the regulatory networks particularly within the neuroectoderm that provide insights about whether there are distinct regulatory programs associated with all bilaterian brain regionalization programs that might support a common origin. It is worth noting, that efforts need to be made to work towards a consensus about what would be considered conserved vs. not conserved at network levels, and that it would be preferable to have those discussions prior to interpreting data. Regardless, pairing efforts to understand inductive programs and regulatory networks around regionalization genes will facilitate efforts to resolve the debate about the origin of brains/CNSs.

## Methods

### Animal care, microinjection, and fixation

*Nematostella* polyps were maintained in *Nematostella* media (12ppt artificial sea water (Instant Ocean)), maintained in the dark at 17 °C, and fed artemia four times per week. One week prior to spawning induction polyps were fed oyster and the *Nematostella* media was replaced. Microinjections were performed as previously described on a Nikon SMZ1270 stereo scope^52,53^. Embryos were raised at either 17 °C or 22 °C to the desired stage. Embryos were fixed and stored as previously described^53^.

### shRNA, mRNA, pharmacological treatments, and plasmid injections

shRNAs were designed and synthesized as previously described^54^, then stored at -80 in single use aliquots. shRNA sequences and primers used to generate them can be found in (Table S3). A previously published scrambled sequence shRNA was used as the control for all shRNA injections^54^. Gene knockdown was confirmed through in situ hybridization and/or qPCR. *Nvsix3/6:venus* was generated by subcloning the *Nvsix3/6* coding sequence into pENTR/D TOPO (ThermoFisher Scientific) using published primers previously used to PCR amplify *Nvsix3/6* and synthesize *Nvsix3/6* mRNA^43^*.* A 3′ Venus tag was added by recombining the *Nvsix3/6* coding sequence into the pSPE3-R-Venus destination vector using the Gateway LR cloning reaction (ThermoFisher Scientific). mRNA was synthesized and injected at 300 ng/µL using previously described methods. Pharmacological treatments were performed either from 3 h post fertilization (hpf) until 24 hpf at 22 °C or from 24 to 48 hpf at 17 °C. Stocks of the Wnt agonist 1-Azakenepaullone (Sigma A3734) or Wnt antagonist iCRT14 (Sigma SML0203) were generated by dissolving each compound in DMSO at 10 mg/mL. Control embryos were treated with DMSO equal to the volume of stock compounds added to the *Nematostella* medium. Embryos were washed with fresh *Nematostella* media prior to fixation.

To synthesize the *Nvserum amyloid A-like∆Nvsix3/6::mcherry* and the *Nvfoxq2d∆Nvsix3/6::mcherry,* the predicted Nvsix3/6 binding motifs were first identified using http://cisbp.ccbr.utoronto.ca/TFTools.php and the published binding domain from Sebés et al. 2018 in the known enhancer sequences of *Nvserum amyloid A-like* and *Nvfoxq2d*^44,46,55^. To remove the predicted *Nvsix3/6* binding sites 1 and 2 in the *Nvserum amyloid A* promoter, the enhancer sequences upstream and downstream of predicted binding sites were subcloned into pGEM-T and the enhancer was reconstituted with binding sites absent. To remove the predicted *Nvsix3/6* binding sites in the *Nvfoxq2d* promoter, primers were designed to remove a 279 bp region containing all three *Nvsix3/6* binding domains. Each enhancer, *Nvserum amyloid A-like∆Nvsix3/6* and *Nvfoxq2d∆Nvsix3/6,* was then subcloned into the pNvT-mcherry reporter construct using the PacI and AscI restriction sites to place the enhancer upstream of the *mcherry* coding sequence. Plasmids were injected into wildtype embryos at 60 ng/µL. F0s were lightly fixed, as previously described^56^, and quantified by recording the number of mCherry positive cells at the aboral end at the late gastrula stages.

### In situ hybridization, imaging, and domain quantifications

In situ hybridization was performed using previously published methods^53^. DIC images of *Nematostella* embryos were taken on a Nikon NTi with a Nikon DS-Ri2 color camera using the Nikon Elements software. To quantify domain size, embryos were rotated so that lateral images were acquired at a medial focal plane that allowed identification of pharyngeal ectoderm. Images were then uploaded to Fiji where domain size was measured using the segmented line tool^57^. Three measurements were taken to determine domain size: (1) total size of embryo from pharyngeal ectoderm to future apical tuft, (2) pharyngeal ectoderm to oral most gene expression, and (3) future apical tuft to the most aboral end of gene expression. We then used these three measurements to determine the start and end of the expression domains along the oral-aboral axis of the embryo, recorded as a percentage of the embryo. The start of the expression domain was calculated by dividing measurement 2 by measurement 1, which was then multiplied by 100. The aboral end of the expression domain was calculated by dividing measurement 3 with measurement 1, multiplying the value by 100, and then subtracted by 100 which demarcates the end of the expression domain.

### Statistical analysis

Statistical analyses were performed with Microsoft excel (version 16). Data are presented here as the mean value calculated with error bars representing a 95% confidence interval. Statistical significance was calculated using a two-tailed student t-test assuming unequal variance. The reported n represents the total number of embryos assessed, but experiments included were repeated a minimum of 2 times with all replicates showing the same results. All raw data available in Supplemental Data Table 1.

### Single cell transcriptomics data processing

Raw sequencing data was processed through the CellRanger 7.0 pipeline using default parameters and aligned to both introns and exons within the *N.vectensis* genome annotation^58^. Counts were then imported in R-studio and processed using the standard Seurat v4 protocol^59^. We filtered out low quality cells with over 10% mitochondrial gene expression and less than 500 genes/cell^47^. Mitochondrial markers were identified using the mitochondrial features listed in Supplemental Table 1. The data was then normalized using the default “LogNormalize” function, 4000 highly variable genes were selected with the “vst” method using the “FindVariableGenes” function, principle components were calculated, and then UMAP graph-based clustering occurred. Clusters with low quality reads were then removed, determined by lower-than-average cell count and the differentially expressed genes found within each low-quality cluster. We then clustered the remaining cells using a low resolution (r = 0.5) to broadly identify published subtypes, ectodermal, and mesoendodermal domain markers using the markers found in Table S2. We then visualized gene expression of potential neural markers using the FeaturePlot function.

## Supplementary Information

Supplementary Figure 1.

Supplementary Figure 2.

Supplementary Figure 3.

Supplementary Legends.

Supplementary Table 1.

Supplementary Table 2.

Supplementary Table 3.

Supplementary Information.

## Data Availability

Raw quantitative data included within the supplemental materials. The datasets generated and/or analysed during the current study are available in the NCBI GEO (Gene Expression Omnibus) repository (https://www.ncbi.nlm.nih.gov/geo/) under the series number GSE218419.
