# Supplementary figures and images for: The brain regulatory program predates central nervous system evolution
Source: Sci Rep. 2023 May 27;13:8626. doi: 10.1038/s41598-023-35721-4 (PMC10224969; doi:10.1038/s41598-023-35721-4)

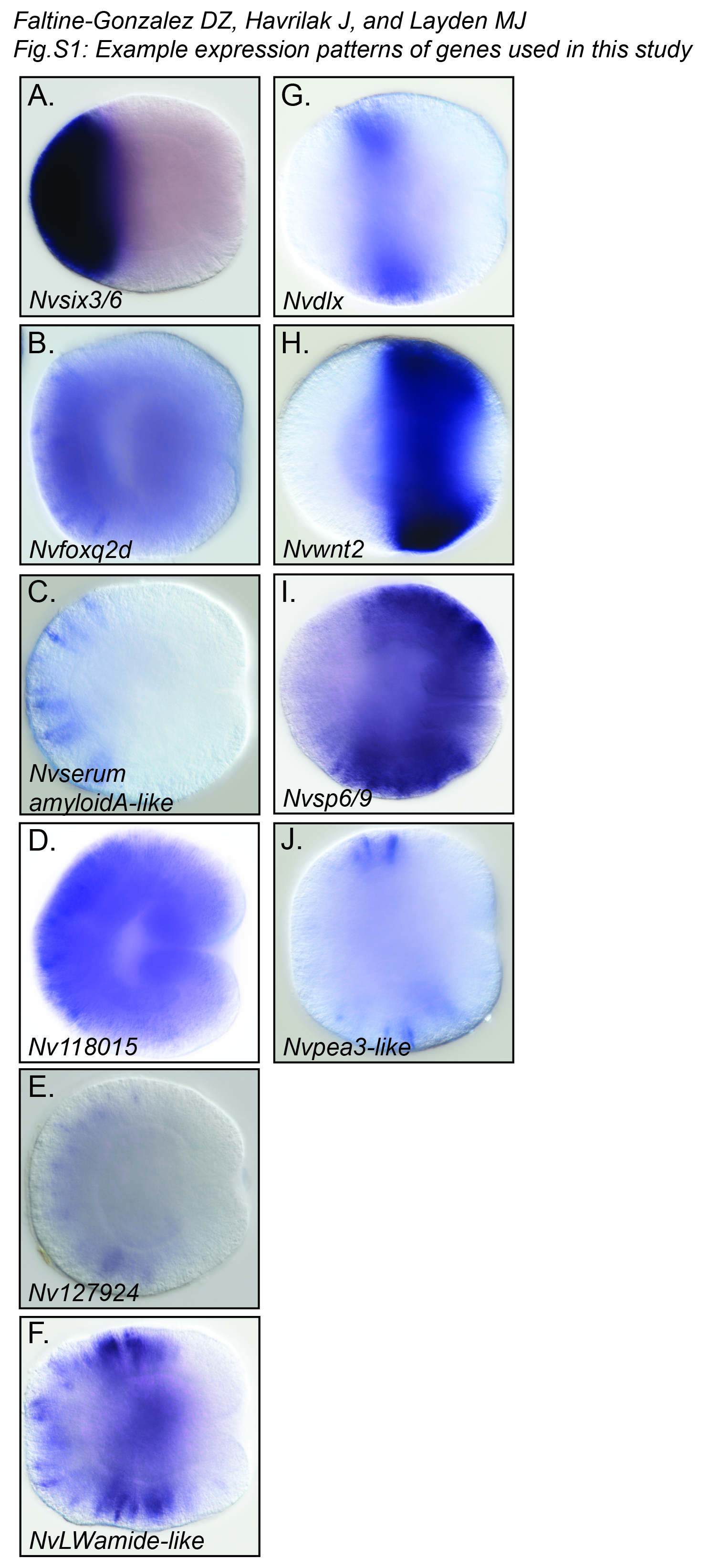

Supplement: Supplementary file 1 — Supplementary Figure 1. [file 41598_2023_35721_MOESM1_ESM.tif]

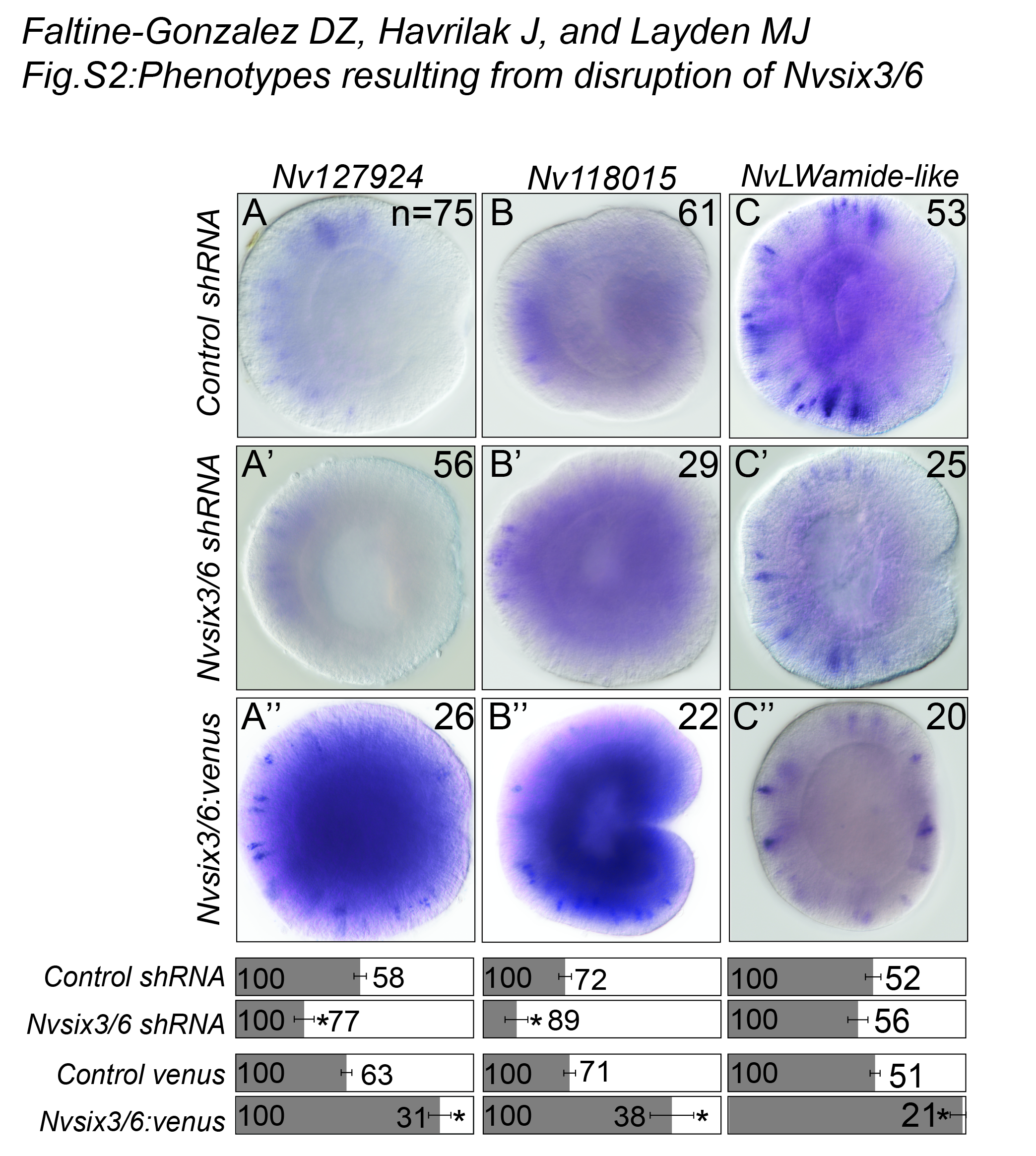

Supplement: Supplementary file 2 — Supplementary Figure 2. [file 41598_2023_35721_MOESM2_ESM.tif]

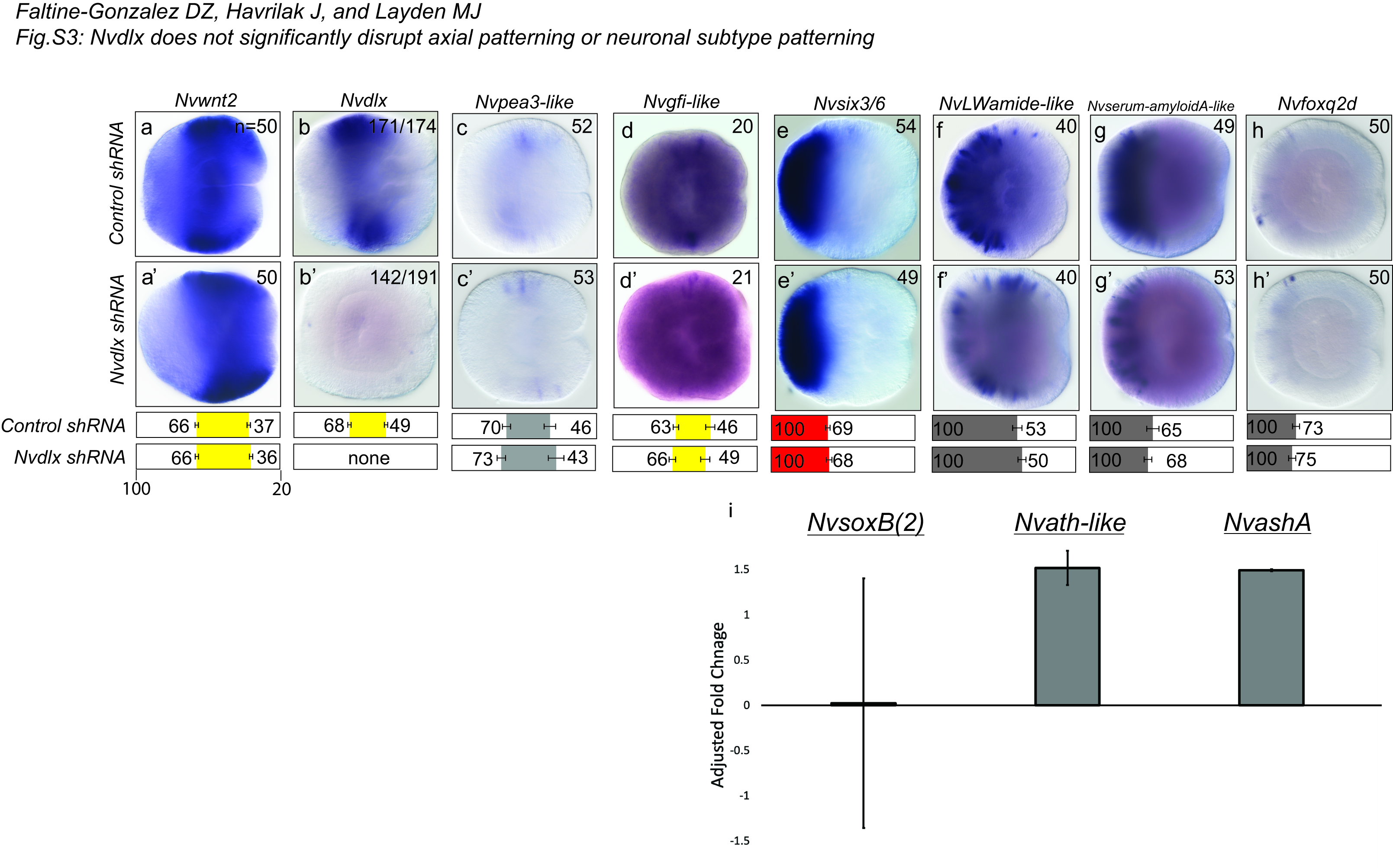

Supplement: Supplementary file 3 — Supplementary Figure 3. [file 41598_2023_35721_MOESM3_ESM.tif]
