# Supplementary material for: The brain regulatory program predates central nervous system evolution
Source: Sci Rep. 2023 May 27;13:8626. doi: 10.1038/s41598-023-35721-4 (PMC10224969; doi:10.1038/s41598-023-35721-4)
Supplement: Supplementary file 4 — Supplementary Legends. [file 41598_2023_35721_MOESM4_ESM.pdf]

Supplemental Figures Faltine-Gonzalez, Havrilak, Layden

Title: The brain regulatory program predates central nervous system evolution.

Authors: Dylan Faltine-Gonzalez[1], Jamie Havrilak[1], Michael J Layden[1†]

Affiliations: [1] Lehigh University, Department of Biological Sciences, Bethlehem, PA USA, † indicates corresponding author

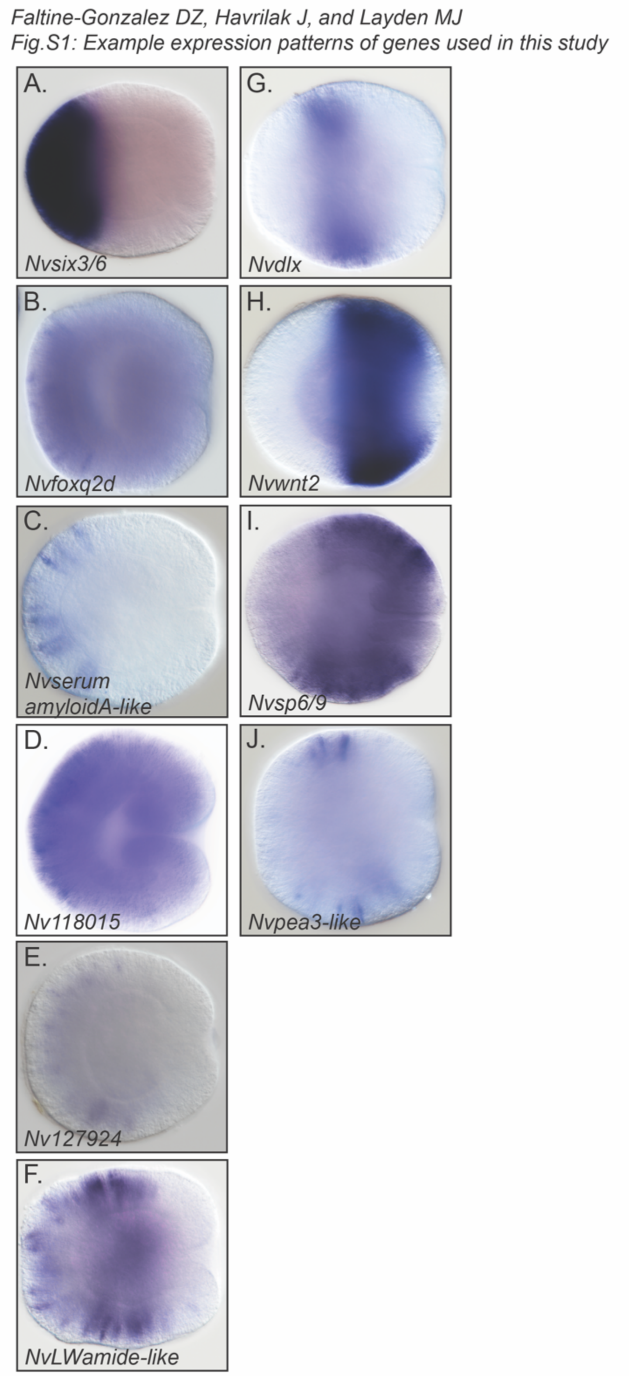

Fig.S1. Example expression patterns of genes used in this study. (A-F) Aboral genes. **(G-J)** Trunk genes.

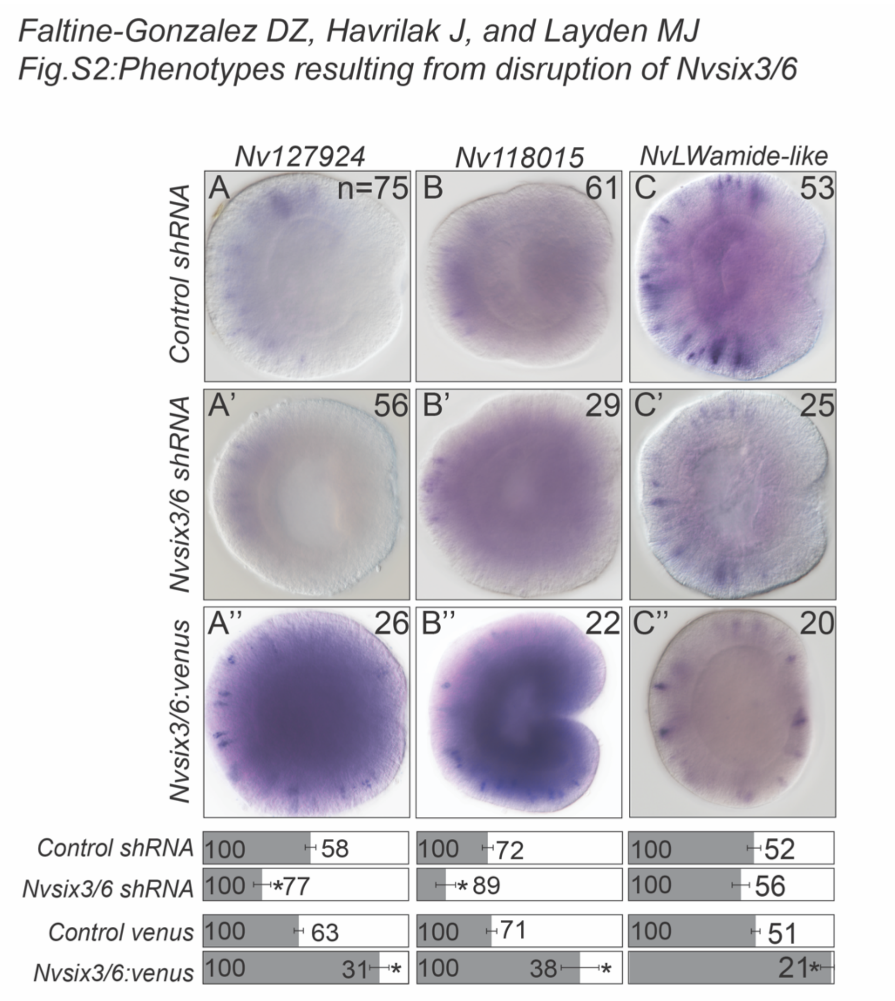

Fig.S2. Phenotypes resulting from disruption of *Nvsix3/6.* (A-C) Control sRNA injected animals. **(A'-C')** *Nvsix3/6* shRNA injected animals. **(A''- C'')** *Nvsix3/6:venus* mRNA injected animals treated. In all images, aboral is to the left and oral is to the right. Quantification of percent embryo length (PEL) positions for the aboral (left-boundary) and oral (right-boundary) expression limits for each treatment and their control are shown below images. Bars represent 95% confidence interval. * indicates that treated values are statistically different ($p \leq 0.05$) from controls and that the 95% confidence intervals for the values do not overlap, and † indicates that treated values are statistically different ($p \leq 0.05$) from controls, but that 95% confidence intervals overlap.

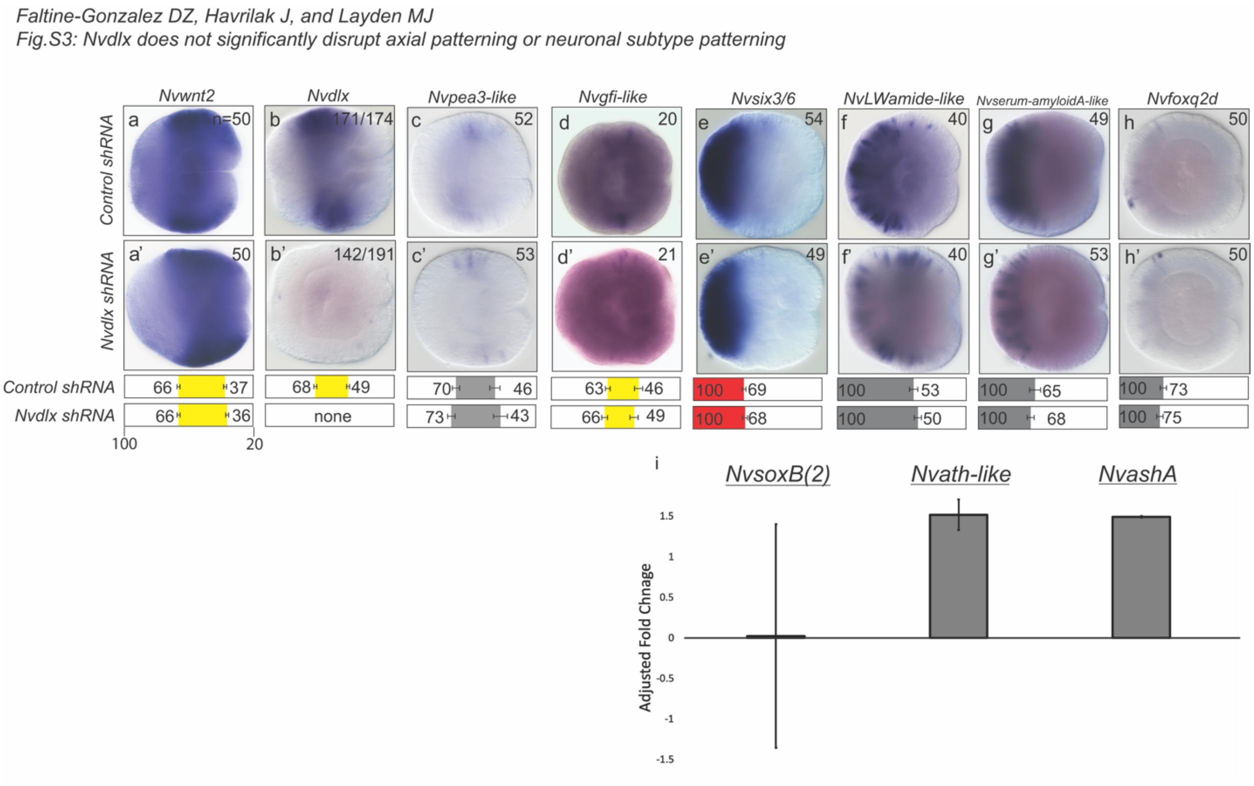

Fig.S3. ***Nvdlx*** **does not significantly disrupt axial patterning or neuronal subtype patterning. (a-h)** Control sRNA injected animals. **(a'-h')** *Nvdlx* shRNA injected animals. **(i)** qPCR in *Nvwnt2* shRNA injected animals. In all images, aboral is to the left and oral is to the right. Quantification of percent embryo length (PEL) positions for the aboral (left-boundary) and oral (right-boundary) expression limits for each treatment and their control are shown below images. Bars represent 95% confidence interval. * indicates that treated values are statistically different ($p \leq 0.05$) from controls and that the 95% confidence intervals for the values do not overlap, and † indicates that treated values are statistically different ($p \leq 0.05$) from controls, but that 95% confidence intervals overlap.

Table S1. Homologs of regionally expressed genes.

This table contains the reported homologs compared across species in the literature. The reference each homolog was identified in is listed in parentheses.

Table S2. Known cluster markers used in previous studies

Marker genes used to identify clusters in previous studies are shown.

Table S3. Reagents used in this study

Description of primers used to generate shRNAs and transgenic constructs used in this study.

Data Table S1. Domain measurements for domain genes and neuronal genes acquired in this study.

List of raw aboral and oral domain limits measured in wild type, control injected, shRNA injected, mRNA injected, and pharmacologically treated animals in this study.
