# Supplementary material for: The brain regulatory program predates central nervous system evolution
Source: Sci Rep. 2023 May 27;13:8626. doi: 10.1038/s41598-023-35721-4 (PMC10224969; doi:10.1038/s41598-023-35721-4)
Supplement: Supplementary file 5 — Supplementary Table 1. [file 41598_2023_35721_MOESM5_ESM.pdf]

Vertebrata	Urochordata	Hemiochordata	Echinodermata	Anthropoda (D.melanogaster)	Anthropoda (T.castaneum)	Annelida	Brachiopodia	Cnidaria
six3 (5,17)	six3/6 (20)	six3 (8)	Sp-six3 (12, 15, 23)	optix (5,12)	Tc-six3 (60, 12)	six3 (5, 12, 32)	Tt-six3/6 (31)	Nvsix3/6 (39,42,43)
dlx2 (8)	Amphi-dll (5)	dlx (8)	Sp-dlx (12, 23)	dll (24)	Tc-dll (60)	dll (8)	-	Nvdlx (39)
pax6 (8)	Pax4/6 (20)	pax6 (8)	Sp-pax6 (19)	pax6(eyeless) (8)	Tc-pax6 (60)	-	-	-
rx (8)	rx (20)	rx (8)	Sp-rx (19)	drx (8)	Tc-rx (60)	rx (32)	-	Nvrx (39, 41)
gbx (2,5)	gbx (2, 20, 21)	gbx (2, 8)	-	unpg/gbx (5)	-	gbx (5, 22)	-	-
irx (2,5)	irxB (2, 20)	irx (2, 8)	Sp-irxA (19)	irx (5)	Tc-irx (60)	irx (32)	-	Nvirx (39)
otx (2, 5, 8)	otx2 (2, 20)	otx (2, 8)	Sp-otx (19)	otd (5)	Tc-otd (60, 18, 19)	otx (5,12, 22,32)	Tt-otx (2,31)	-
otp (8)	otp (20)	otp (8)	-	-	-	otp (32)	Tt-otp (31)	Nvotp (41)
fezf (2, 5, 11)	fezf (2, 20)	fezf (8)	Sp-fez (29)	earmuff (5, 11)	Tc-fez (60)	fezf (32)	Tt-fez (31)	Nvfez (44)
pax2/5/8 (5)	pax2/5/8 (20)	-	-	pax2/5/8 (5, 27)	-	pax2/5/8 (5)	Tt-pax2/5/8 (31)	Nvpax2/5/8 (40)
-	-	foxq2-1/foxq2-2 (8)	Sp-foxq2 (19)	-	Tc-foxQ2 (60)	foxq2 (32)	Tt-foxq2 (31)	Nvfoxq2d (44, 46)

References: Numbers correspond to reference number in main text.

2. Martín-Durán, J. M. & Hejnol, A. A developmental perspective on the evolution of the nervous system. *Dev Biol* 475, 181–192 (2019).

5. Holland, L. Z. *et al.* Evolution of bilaterian central nervous systems: a single origin? *EvoDevo* 4, 1–1 (2013).

8. Lowe, C. J. *et al.* Anteroposterior Patterning in Hemichordates and the Origins of the Chordate Nervous System. *Cell* 113, 853–865 (2003).

11. Irimia, M. *et al.* Conserved developmental expression of Fezf in chordates and Drosophila and the origin of the Zona Limitans Intrathalamica (ZLI) brain organizer. *Evodevo* 1, 7 (2010).

12. Steinmetz, P. R. *et al.* Six3 demarcates the anterior-most developing brain region in bilaterian animals. *EvoDevo* 1, 14 (2010).

15. Petersen, C. P. & Reddien, P. W. Wnt Signaling and the Polarity of the Primary Body Axis. *Cell* 139, 1056–1068 (2009).

17. Lagutin, O. V. *et al.* Six3 repression of Wnt signaling in the anterior neuroectoderm is essential for vertebrate forebrain development. *Gene Dev* 17, 368–379 (2003).

18. Schinko, J. B. *et al.* Divergent functions of orthodenticle, empty spiracles and buttonhead in early head patterning of the beetle Tribolium castaneum (Coleoptera). *Dev Biol* 317, 600–613 (2008).

19. Range, R. C. & Wei, Z. An anterior signaling center patterns and sizes the anterior neuroectoderm of the sea urchin embryo. *Development* 143, 1523–1533 (2016).

20. Albuixech-Crespo, B. *et al.* Molecular regionalization of the developing amphioxus neural tube challenges major partitions of the vertebrate brain. *Plos Biol* 15, e2001573 (2017).

21. Castro, L. F. C., Rasmussen, S. L. K., Holland, P. W. H., Holland, N. D. & Holland, L. Z. A Gbx homeobox gene in amphioxus: Insights into ancestry of the ANTP class and evolution of the midbrain/hindbrain boundary. *Dev Biol* 295, 40–51 (2006).

22. Steinmetz, P. R. H., Kostyuchenko, R. P., Fischer, A. & Arendt, D. The segmental pattern of otx, gbx, and Hox genes in the annelid Platynereis dumerilii: The segmental pattern of otx, gbx, and Hox genes. *Evol Dev* 13, 72–79 (2011).

23. Howard-Ashby, M. *et al.* Identification and characterization of homeobox transcription factor genes in Strongylocentrotus purpuratus, and their expression in embryonic development. *Dev Biol* 300, 74–89 (2006).

24. Panganiban, G. Distal-less function during Drosophila appendage and sense organ development. *Dev Dynam* 218, 554–562 (2000).

27. Czerny, T., Bouchard, M., Kozmik, Z. & Busslinger, M. The characterization of novel Pax genes of the sea urchin and Drosophila reveal an ancient evolutionary origin of the Pax2/5/8 subfamily. *Mech Develop* 67, 179–192 (1997).

29. Yaguchi, S. *et al.* Fez function is required to maintain the size of the animal plate in the sea urchin embryo. *Development* 138, 4233–4243 (2011).

31. Santagata, S., Resh, C., Hejnol, A., Martindale, M. Q. & Passamaneck, Y. J. Development of the larval anterior neuronal domains of Terebratalia transversa (Brachiopoda) provides insights into the diversification of larval apical organs and the spiralian nervous system. *Evodevo* 3, 3 (2012).

32. Marlow, H. *et al.* Larval body patterning and apical organs are conserved in animal evolution. *Bmc Biol* 12, 7 (2014).

39. Marlow, H., Matus, D. Q. & Martindale, M. Q. Ectopic activation of the canonical wnt signaling pathway affects ectodermal patterning along the primary axis during larval development in the anthozoan Nematostella vectensis. *Dev Biol* 380, 324–334 (2013).

40. Matus, D. Q., Pang, K., Daly, M. & Martindale, M. Q. Expression of Pax gene family members in the anthozoan cnidarian, Nematostella vectensis. *Evol Dev* 9, 25–38 (2007).

41. Mazza, M. E., Pang, K., Reitzel, A. M., Martindale, M. Q. & Finnerty, J. R. A conserved cluster of three PRD-class homeobox genes (homeobrain, rx and orthopedia) in the Cnidaria and Protostomia. *Evodevo* 1, 3 (2010).

42. Sinigaglia, C., Busengdal, H., Leclère, L., Technau, U. & Rentzsch, F. The Bilaterian Head Patterning Gene six3/6 Controls Aboral Domain Development in a Cnidarian. *Plos Biol* 11, e1001488 (2013).

43. Leclère, L., Bause, M., Sinigaglia, C., Steger, J. & Rentzsch, F. Development of the aboral domain in Nematostella requires β-catenin and the opposing activities of Six3/6 and Frizzled5/8. *Dev Camb Engl* 143, 1766–1777 (2016).

44. Layden, M. J. *et al.* MAPK signaling is necessary for neurogenesis in Nematostella vectensis. *Bmc Biol* 14, 61 (2016).

46. Busengdal, H. & Rentzsch, F. Unipotent progenitors contribute to the generation of sensory cell types in the nervous system of the cnidarian Nematostella vectensis. *Dev Biol* 431, 59–68 (2017).

60. Posnien, N., Koniszewski, N.D.B., Hein, H.J., & Bucher, G. Candidate Gene Screen in the Red Flour Beetle Tribolium Reveals Six3 as Ancient Regulator of Anterior Median Head and Central Complex Development. *PLoS Genet.* 7, e1002416 (2011).
