# Supplementary material for: The brain regulatory program predates central nervous system evolution
Source: Sci Rep. 2023 May 27;13:8626. doi: 10.1038/s41598-023-35721-4 (PMC10224969; doi:10.1038/s41598-023-35721-4)
Supplement: Supplementary file 6 — Supplementary Table 2. [file 41598_2023_35721_MOESM6_ESM.docx]

**Table S1.**

|  | Cluster | Genome Identification | Annotation |
| --- | --- | --- | --- |
| 1 | Trunk Ectoderm | NV2g003168000.1 | Nvwnt2 |
| 2 | Aboral Ectoderm | NV2g011441000.1 | Nvsix3/6 |
| 3, 8 | Mesoendoderm 1, Mesoendoderm 2 | NV2g000472000.1 | NvsnailA |
| 4 | Pharyngeal Ectoderm | NV2g011441000.1 | NvfoxA |
| 5, 7, 11, 13 | Cnidocytes 1, Cnidocytes 2, Cnidocytes 3, Cnidocyte 4 | NV2g010686000.1 | Nvncol3 |
|  |  | NV2g005200001.1 | NvNgal |
|  |  | NV2g011820000.1 | Nvncol1 |
| 6 | Gland | NV2g012902000.1 | Nvmucin |
| 9, 12, 14 | Neuron 1 | NV2g000252000.1 | Nvelav |
|  |  | NV2g023192000.1 | Nvlwamide |
|  |  | NV2g009665000.1 | Nvasha |
| 10 | Progenitors | NV2g006608000.1 | Nvath-like |
|  |  | NV2g018827000.1 | Nvmyc1 |
|  |  | NV2g000437000.1 | Nvnanos |
|  |  |  |  |
|  |  |  |  |
|  |  |  |  |
| **Mitochondrial Genes** | |  |  |
| NV2g000053000.1 | ATP Synthase Mitochondrial F1 Complex assembly factor 1 | | |
| NV2g025931000.1 | NADH-ubiquinone oxidoreducatase chain 2 | |  |
| NV2g006232000.1 | COX assembly mitochondrial protein homolog | |  |
| NV2g024053000.1 | Cytochrome c oxidase subunit 5A, mitochondrial | |  |
| NV2g007852000.1 | ATP Synthase Mitochondrial F1 Complex assembly factor 2 | | |
| NV2g000772000.1 | Cytochrome c oxidase subunit 5B | |  |
| NV2g001453000.1 | Succinate--CoA ligase [GDP-forming] subunit beta, mitochondrial | | |
| NV2g002690000.1 | Translation factor GUF1 homolog, mitochondrial | |  |
| NV2g002540000.1 | Heat shock protein 75 kDa, mitochondrial | |  |
| NV2g002424000.1 | 39S ribosomal protein L22, mitochondrial | |  |
|  |  |  |  |
